# An investigation into the beneficial effects and molecular mechanisms of humic acid on foxtail millet under drought conditions

**DOI:** 10.1371/journal.pone.0234029

**Published:** 2020-06-02

**Authors:** Jie Shen, Meijun Guo, Yuguo Wang, Xiangyang Yuan, Shuqi Dong, Xi-e Song, Pingyi Guo

**Affiliations:** Department of Agronomy, College of Agriculture, Shanxi Agricultural University, Taigu County, Jinzhong, Shanxi, China; Institute for Sustainable Plant Protection, C.N.R., ITALY

## Abstract

The aim of this study was to determine the effects and underlying molecular mechanisms of humic acid (HA) on foxtail millet (*Setaria italica* Beauv.) under drought conditions. The rainless climate of the Shanxi Province (37°42'N, 112°58'E) in China provides a natural simulation of drought conditions. Two foxtail millet cultivars, Jingu21 and Zhangza10, were cultivated in Shanxi for two consecutive years (2017–2018) based on a split-plot design. Plant growth, grain quality, and mineral elements were analyzed in foxtail millet treated with HA (50, 100, 200, 300, and 400 mg L^-1^) and those treated with clear water. Transcriptome sequencing followed by bioinformatics analysis was performed on plants in the normal control (CK), drought treatment (D), and drought + HA treatment (DHA) groups. Results were verified using real-time quantitative PCR (RT-qPCR). HA at a concentration of 100–200 mg L^-1^ caused a significant increase in the yield of foxtail millet and had a positive effect on dry weight and root-shoot ratio. HA also significantly increased P, Fe, Cu, Zn, and Mg content in grains. Moreover, a total of 1098 and 409 differentially expressed genes (DEGs) were identified in group D vs. CK and D vs. DHA, respectively. A protein-protein interaction network and two modules were constructed based on DEGs (such as SETIT_016654mg) between groups D and DHA. These DEGs were mainly enriched in the metabolic pathway. In conclusion, HA (100 mg L^-1^) was found to promote the growth of foxtail millet under drought conditions. Furthermore, SETIT_016654mg may play a role in the effect of HA on foxtail millet via control of the metabolic pathway. This study lays the foundation for research into the molecular mechanisms that underlie the alleviating effects of HA on foxtail millet under drought conditions.

## Introduction

Foxtail millet (*Setaria italica* Beauv.) is a grain crop that grows in arid and semi-arid regions. It is an important crop in many areas of Africa and Asia due to its ability to grow in harsh environments [1], and is the dominant food crop in many provinces of China, including Shanxi [1]. Shanxi Province has a typical continental arid climate with annual rainfall ranging between 400–650 mm, which is below standard irrigation conditions [2]. It is therefore of great practical importance to investigate the drought-resistant growth mechanisms of foxtail millet and identify ways to increase its yield in arid and semi-arid conditions.

Humic Acid (HA) is a natural organic polymer compound and a major component of humus, which plays an important role in crop quality, yield, and stress resistance [3, 4]. According to recent reports, HA affects plant quality in two major ways: helping to resist stress by controlling the amount of reactive oxygen species present [5], and promoting growth, photosynthesis, nitrogen assimilation, and amino acid metabolism [4, 6]. Parađiković et al. found that using a mixture of biostimulants containing HA increased the yield of yellow pepper crops. Peptides and amino acids contained in this mixed preparation promoted high temperature resistance in the peppers, and stimulated root growth and development, while vitamins and HA supported fruit growth [7]. A study showed that high concentrations of HA had a significant positive effect on the morphological characteristics of cucumber, including plant height, leaf number, and fresh weight and yield. Additionally, the percentage of total chemical components [nitrogenium (N), phosphorus (P), potassium kalium (K), calcium (Ca), and magnesium (Mg)] in the leaves of cucumber plants increased as HA concentration increased [8]. Maji et al. showed that HA facilitates plant growth by improving the microbial community structure of soil and increasing mycorrhizal colonization in the roots of *Pisum sativum* [9], while another study found that foliar-applied HA improved dry weight and mineral nutrient uptake [such as manganese (Mn), copper (Cu), zinc (Zn), and calcium (Ca)] of maize [10]. HA has also been shown to stimulate nitrogen assimilation and amino acid metabolism in maize at both the physiological and molecular level [6]. Olaetxea et al. demonstrated that root hydraulic conductivity and aquaporin-related gene expression are important for plant shoot outgrowth induced by HA [11], and Kuşvuran et al. described the effects of different HA treatments on yield and performance in common millet. Together, these results indicate that HA treatment can significantly improve plant yield and quality [12]. However, despite all of these promising reports, the effects of HA on foxtail millet, and the underlying molecular mechanisms, remain unknown

In the present study, we utilized the natural drought conditions in Shanxi Province to investigate the effects of HA on crop yield and quality of foxtail millet. The optimal HA concentration was determined by measuring growth and nutritional indicators in foxtail millet samples after different periods of HA treatment. Moreover, high-throughput sequencing analysis was used to assess changes in gene expression related to photosynthetic assimilation and nutrient indicators. This allowed the molecular mechanisms that underlie the effects of HA on dry matter and nutrient accumulation in foxtail millet to be further elucidated.

## Materials and methods

### Test material

Test materials were selected from ordinary high-quality foxtail millet Jingu21 (Shanxi Academy of Agricultural Sciences Economic Crops Research Institute, China) and hybrid high-yield foxtail millet Zhangza10 (Zhangjiakou City Academy of Agricultural Sciences, Hebei Province, China).

### Field experiments

From 2017 to 2018, field experiments were conducted at the agricultural research station of Shanxi Agricultural University (37°42'N, 112°58'E). This region is dry and has very little rainfall, thus naturally simulating a drought environment. Crops from the previous season were not foxtail millet, and continuous cropping was therefore avoided.

A split-plot design was adopted for this study. Briefly, clear water was used as a blank control for the main plot, while HA at concentrations of 50, 100, 200, 300, and 400 mg L^-1^ (T1, T2, T3, T4, and T5, respectively) was used for the secondary plot. Both water and HA were sprayed onto the leaves of the foxtail millet at the jointing and filling stages (800 L ha^-1^). The experiment was repeated three times in all 36 districts (10 m^2^ district^-1^).

### Plant quality evaluation

The foxtail millet was harvested on October 13, 2017, and October 1, 2018. The ear, stem, leaf, and root from three representative mature plants were baked at 105°C for 30 min, followed by 80°C for 24–48 h, then the dry weight was measured. Plant height, stem diameter, ear length, ear diameter, ear yardage, and 1000-grain weight were measured on three representative mature plants. Grain ears were manually threshed, shelled, comminuted (0.5 mm sieve), and dried. Protein content was determined using an MPA Fourier transform near-infrared spectrometer (Bruke, Germany). Total P and K content was determined via the molybdenum antimony colorimetric method and flame photometry. Presence of mineral elements, such as iron (Fe), Mn, Cu, Zn, Ca, and Magnesium (Mg), was determined using the diethylenetriaminepentaacetic acid (DTPA) extraction inductively-coupled plasma spectroscopy (ICP) method.

### HA treatment evaluation based on the membership function method

The yield and quality of foxtail millet Jingu21 and Zhangza10 after treatment with different HA concentrations were comprehensively analyzed using the membership function method in fuzzy mathematics [13]. The membership function formula is:

$$U\;\left(X_{i}\right)=\left(Xi‑X_{min}\right)/\left(X_{max}‑X_{min}\right)\;\left(indicator\;trait\;positively\;correlates\;with\;nutrient\;uptake\;and\;yield\right)$$

$$U\;\left(X_{i}\right)=1‑\left(Xi‑X_{min}\right)/\left(Xmax‑X_{min}\right)\;\left(indicator\;trait\;negatively\;correlates\;with\;nutrient\;uptake\;and\;yield\right)$$

where U (X_i_) is the membership function value; X_i_ is the measured value of an index at each processing level; X_max_ and X_min_ are the maximum and minimum values within an indicator at all processing levels, respectively.

### Grouping for molecular analysis

Leaves from 3–5 leaf potted foxtail millet seedlings were sequenced to ensure accuracy of the test. Sequencing samples (Jingu21) were divided into three groups; normal control group (CK), drought treatment group (D), and drought + HA treatment group (DHA). Plants in the CK group were cultured under normal conditions. Culture conditions for plants in groups D and DHA were in accordance with field trials. The optimal HA concentration identified during field trials was used as the default concentration for HA treatment (100 mg L^-1^). Seedlings in groups D and DHA were harvested and analyzed after five days of drought.

### Transcriptome sequencing

Total RNA was extracted from samples from all groups using the TRIzol-based method (RNAiso Plus, TaKaRa, 9109). RNA quantification and purity were determined using diethyl phosphorocyanidate (DEPC) H_2_O as the blank control. Transcriptome sequencing was then performed based on Illumina high throughput sequencing. The design and detailed operation for sequencing were validated by Beijing Novogene Technology Co., Ltd (Project Number: P101SC18122767-01). Sequencing data were retained for subsequent analysis.

### Quality control and preprocessing

Quality control and preprocessing were performed on the original sequencing data. A clean read was obtained by filtering joint and low-quality sequences. Quality control of the clean reads was performed using fastqc (version: 0.11.5, http://www.bioinformatics.babraham.ac.uk/projects/fastqc/) [14]. TopHat software (version: 2.1.0, http://ccb.jhu.edu/software/tophat/) [15] was used to locate clean reads on the reference genome Setaria_italica_v2.0 (version: Ensembl Plants) [16] for foxtail millet. Finally, the featureCounts tool (version: 1.6.0, http://subread.sourceforge.net/) [17] was used to annotate the samples with a Gens genome annotation file (Ensembl Plants), and read information for each gene alignment was obtained.

### DEGs analysis

According to the RNA read count data, the read count was pretreated using the TMM (trimmed mean of M values) normalization method within the edgeR package in R software [18, 19]. DEGs between D vs. CK and D vs. DHA were then revealed using the quasi-likelihood (QL) F-test in the edgeR package. Results were visualized via a heat map using pheatmap software (https://cran.r-project.org/web/packages/pheatmap). DEGs in both the D vs. CK and D vs. DHA groups were then visualized using a Venn diagram generated using VENNY software (http://bioinfogp.cnb.csic.es/tools/venny/index.html) [20].

### PPI network construction and module analysis

Protein interaction information was obtained according to the search tool for the retrieval of interacting genes (STING) database (version: 11.0, http://www.string-db.org/) [21], and PPI pairs among DEGs between groups D and DHA were predicted with median confidence (score) = 0.4. Next, a PPI network was constructed using Cytoscape software (version: 3.7.0, http://www.cytoscape.org/) [22], and molecular complex detection (MCODE, Version1.5.1 http://apps.cytoscape.org/apps/MCODE) [23], a plug-in of Cytoscape software, was used to screen significantly enriched modules from the PPI network with a module score ≥ 4.

### Pathway enrichment analysis of the DEGs

KEGG (Kyoto Encyclopedia of Genes and Genomes) pathway [24] enrichment analyses of DEGs between groups D and DHA were performed using KOBAS software (version: 3.0, http://kobas.cbi.pku.edu.cn/) [25]. *p* < 0.05 was considered the cut-off for significant enrichment.

### Real-time quantitative PCR

RT-qPCR was used to verify DEGs obtained in this study. Expression of SETIT_009509mg, SETIT_021707mg, SETIT_016840mg, SETIT_015030mg, SETIT_004913mg, and SETIT_016654mg was assessed by qPCR. Briefly, total RNA was extracted from samples from each group (CK1, CK2, CK3, D1, D2, D3, DHA1, DHA2, and DHA3) using TRIzol reagent (RNAiso Plus, TaKaRa, 9109) and quantified. Reverse transcription was performed using 5×primeScript RT Master Mix (Perfect Real Time, TAKARA, RR036A). Actin was used as a reference. Primers are listed in S1 Table. Reaction conditions were as follows: 50°C for 3 min, 95°C for 3 min, 40 cycles at 95°C for 10 s, and 60°C for 30 s. A fluorescence signal was recorded at the end of each cycle, and an amplification curve was generated. Relative expression of candidate genes was calculated using the 2^-ΔΔCT^ method [26].

### Statistical analysis

Statistical analysis was carried out using SPSS 16.0 (Inc., Chicago, IL, USA) and GraphPad Prism 5 software (GraphPad Software, San Diego, CA). Results from field trials were processed and plotted using Microsoft Excel 2010, and data are presented as mean ± standard error (SE). RT-qPCR results are presented as mean ± standard deviation (SD). *p* < 0.05 and *p* < 0.01 were the screening criteria for significant and extremely significant differences, respectively.

## Results

### Effect of HA on growth and yield characteristics of foxtail millet

**Fig 1:**
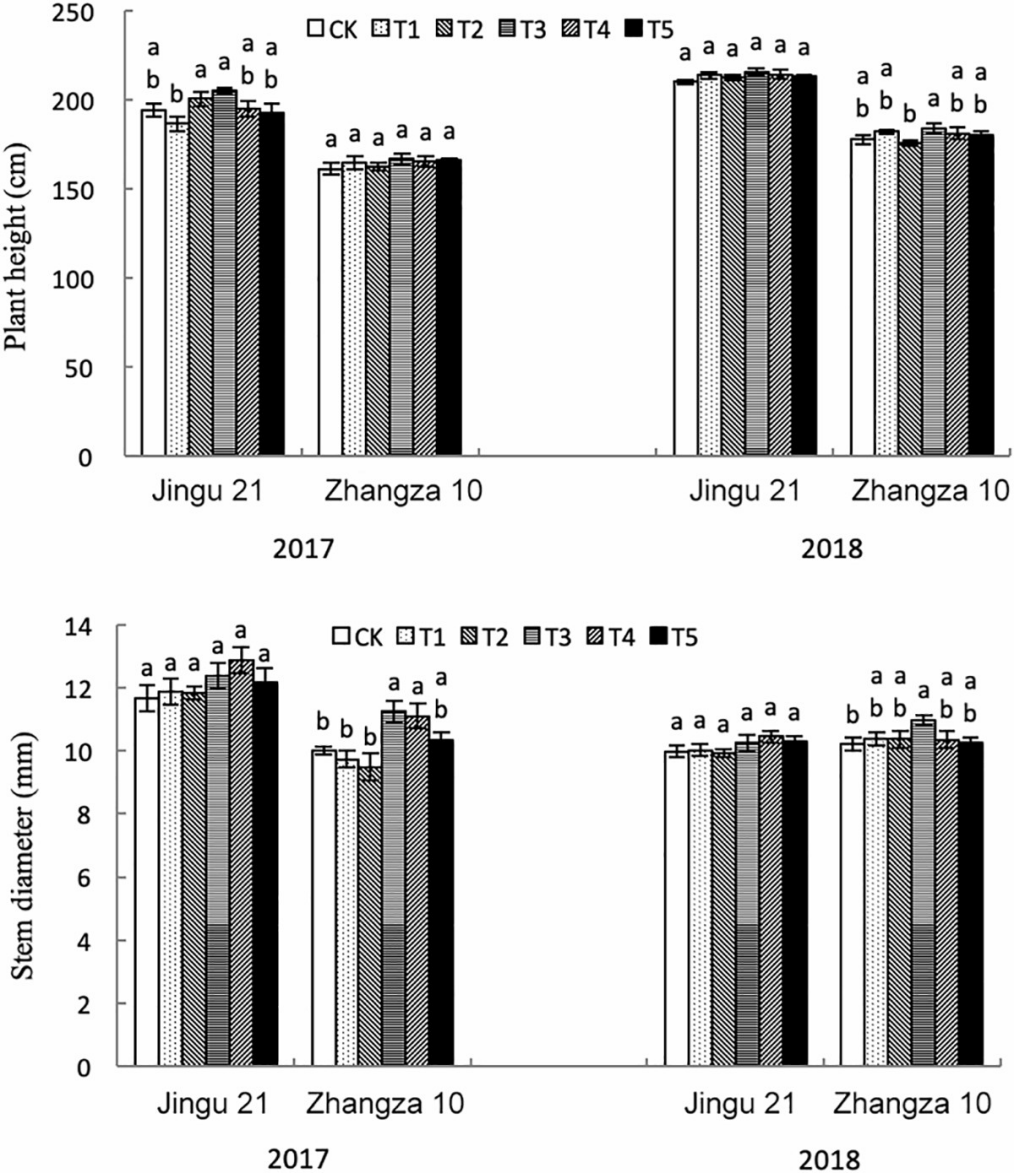
Effect of humic acid on plant height and stem diameter of foxtail millet in the years 2017 and 2018. X-axis represents different cultivars of foxtail millet; y-axis represents different plant traits. CK, T1, T2, T3, T4, and T5 represent different concentrations of HA (0 mg L^-1^, 50 mg L^-1^, 100 mg L^-1^, 200 mg L^-1^, 300 mg L^-1^, and 400 mg L^-1^). Lowercase letters in the same column indicate significant differences at the 0.05 level.

As the HA concentration increased, the stem diameter of Zhangza10 initially increased then decreased, and this was true for samples collected in both 2017 and 2018. The effects of HA on plant height and stem diameter of Jingu21, and plant height of Zhangza10, were not significant (all *p* > 0.05) (Fig 1). In 2017, the underground dry weight of Jingu21 and Zhangza10 initially increased then decreased with increasing HA concentrations (S2 Table). Jingu21 reached its maximum root-to-shoot ratio with T3 in 2017 and 2018, while Zhangza10 reached its maximum root-to-shoot ratio with T2 in both years. These results show that HA at a concentration of 100–200 mg L^-1^ significantly improved the dry weight and root-shoot ratio of foxtail millet.

Moreover, there was an initial increase in ear yardage, ear weight, and yield in both Jingu21 and Zhangza10 as HA concentration increased, but they then decreased (Table 1). Jingu21 had the largest ear diameter and yield with T3 in 2017 and with T2 in 2018, while Zhangza10 had the largest ear diameter and yield with T3 in 2017. In 2018, the maximum ear diameter of Zhangza10 was recorded with T2, while the maximum yield occurred with T3, and this treatment also resulted in the maximum ear length. The yield of Jingu21 increased by 16.96% (two-year average) with T3 and T2, compared to CK. Meanwhile, Zhangza10 yield increased by 14.48% (two-year average) with T3, compared to CK. These results show that HA at a concentration of 100–200 mg L^-1^ causes a significant increase in foxtail millet yield, and has a positive effect on dry weight and root-shoot ratio. The correlation coefficients between growth indicators and yield traits are listed in Table 2. There was a strong negative correlation between yield, plant height and aboveground dry weight (*p* < 0.01), and a positive correlation between yield and root-shoot ratio (*p* < 0.01). Thousand kernel weight showed a strong positive correlation with stem diameter and underground dry weight (*p* < 0.01).

**Table 1:** Effect of HA on yield and yield composition of foxtail millet in 2017 and 2018.

Cultivar	Treatment	Ear length(cm)	Ear diameter(mm)	Ear yardage	Ear weight(g)	1000-grain weight(g)	Yield / kg·ha^-1^
	CK	24.61±0.48^ab^	32.38±0.92^a^	102.3±1.5^b^	21.06±1.07^b^	3.112±0.096^a^	3928.2±28.7^c^
Jingu21	T1	23.67±0.19^b^	31.50±1.16^a^	106.6±2.8^b^	21.75±1.65^b^	3.201±0.018^a^	3595.5±36.8^d^
(2017)	T2	24.50±0.35^ab^	32.33±0.33^a^	117.8±3.5^a^	27.86±1.32^a^	3.264±0.073^a^	3351.7±47.6^e^
	T3	25.00±0.75^ab^	34.23±0.70^a^	114.9±1.6^a^	28.60±2.61^a^	3.252±0.003^a^	4463.5±53.4^a^
	T4	23.83±0.19^ab^	33.78±1.24^a^	105.4±1.2^b^	23.21±0.73^ab^	3.270±0.072^a^	4238.4±63.4^b^
	T5	25.44±0.78^a^	32.82±0.64^a^	105.7±3.2^b^	25.53±1.87^ab^	3.215±0.065^a^	3982.0±43.3^c^
	CK	27.23±0.26^a^	26.53±0.70^ab^	100.6±4.9^b^	25.06±0.37^b^	2.452±0.109^ab^	3741.5±17.8^c^
Jingu21	T1	26.77±0.30^ab^	27.50±0.60^a^	110.3±3.2^ab^	22.48±0.56^c^	2.651±0.029^a^	3572.6±51.1^c^
(2018)	T2	26.13±0.24^b^	28.67±0.68^a^	109.4±4.3^ab^	26.28±0.51^ab^	2.506±0.082^ab^	4500.2±67.2^a^
	T3	26.43±0.20^ab^	28.43±0.60^a^	117.7±4.8^a^	27.30±0.54^a^	2.301±0.124^b^	4158.3±50.5^b^
	T4	25.87±0.37^b^	27.77±0.53^a^	116.6±3.9^a^	20.91±0.42^d^	2.392±0.068^ab^	4195.8±41.6^b^
	T5	26.13±0.39^b^	24.56±0.72^b^	105.4±3.5^ab^	20.37±0.58^d^	2.504±0.101^ab^	3604.7±70.9^c^
	CK	26.08±0.14^a^	29.71±0.98^a^	107.0±4.1^abc^	29.89±3.06^abc^	2.957±0.057^ab^	5160.1±47.9^b^
Zhangza10	T1	24.67±0.44^b^	30.48±0.93^a^	98.9±2.1^c^	31.67±2.91^ab^	2.968±0.062^ab^	5149.4±61.7^b^
(2017)	T2	25.17±0.42^ab^	31.69±0.10^a^	104.4±2.4^bc^	32.22±0.78^a^	3.038±0.023^a^	5414.0±10.1^a^
	T3	25.94±0.24^a^	32.17±1.03^a^	110.3±2.1^ab^	33.67±1.93^a^	3.005±0.024^a^	5447.7±40.2^a^
	T4	25.22±0.49^ab^	30.86±1.01^a^	116.6±3.8^a^	23.67±0.58^c^	2.987±0.031^a^	5123.4±57.2^b^
	T5	25.44±0.31^ab^	31.29±1.11^a^	106.4±2.8^bc^	24.78±2.32^bc^	2.848±0.008^b^	4674.4±72.3^c^
	CK	32.29±0.22^ab^	33.36±0.54^ab^	103.9±3.6^a^	26.94±0.46^b^	2.609±0.042^a^	3828.0±40.0^c^
Zhangza10	T1	31.21±0.41^b^	32.00±0.84^b^	100.9±2.6^a^	24.06±0.88^c^	2.655±0.031^a^	3761.4±76.3^c^
(2018)	T2	30.92±0.66^b^	35.12±0.54^a^	104.9±3.5^a^	28.08±0.52^ab^	2.651±0.035^a^	4401.7±73.5^b^
	T3	33.19±0.75^a^	32.61±0.99^b^	104.9±1.8^a^	29.17±0.55^a^	2.549±0.050^a^	4723.3±89.7^a^
	T4	30.77±0.70^b^	32.90±0.86^ab^	100.0±1.2^a^	22.45±0.42^cd^	2.542±0.028^a^	4601.3±76.6^ab^
	T5	32.08±0.60^ab^	31.54±0.31^b^	101.6±4.0^a^	21.82±0.35^d^	2.636±0.039^a^	3523.1±38.8^d^

CK, T1, T2, T3, T4 and T5 represent HA concentrations (0 mg L^-1^, 50 mg/L, 100 mg L^-1^, 200 mg L^-1^, 300 mg L^-1^ and 400 mg L^-1^); superscript lowercase letters in the same column indicated significant differences at 0.05 level.

**Table 2:** Correlation coefficients between growth indicators and yield traits.

Yield traits	Plant height (cm)	Stem diameter (mm)	Aboveground dry weight (g)	Underground dry weight (g)	Root-shoot ratio (%)
Ear length (cm)	-0.15	-0.37	-0.50*	-0.62**	0
Ear diameter (mm)	-0.50*	0.49*	-0.08	0.41*	0.35
Ear yardage	0.36	0.28	0.35	0.48*	-0.04
Ear weight (g)	-0.47*	-0.15	-0.42*	0.15	0.63**
1000-grain weight (g)	-0.34	0.69**	0.34	0.75**	0.24
Yield (kg·ha^-1^)	-0.64**	-0.2	-0.61**	0.1	0.80**

* and ** denote significant correlation at 5% and 1% probability levels, respectively.

### Seed quality and membership function evaluation

**Fig 2:**
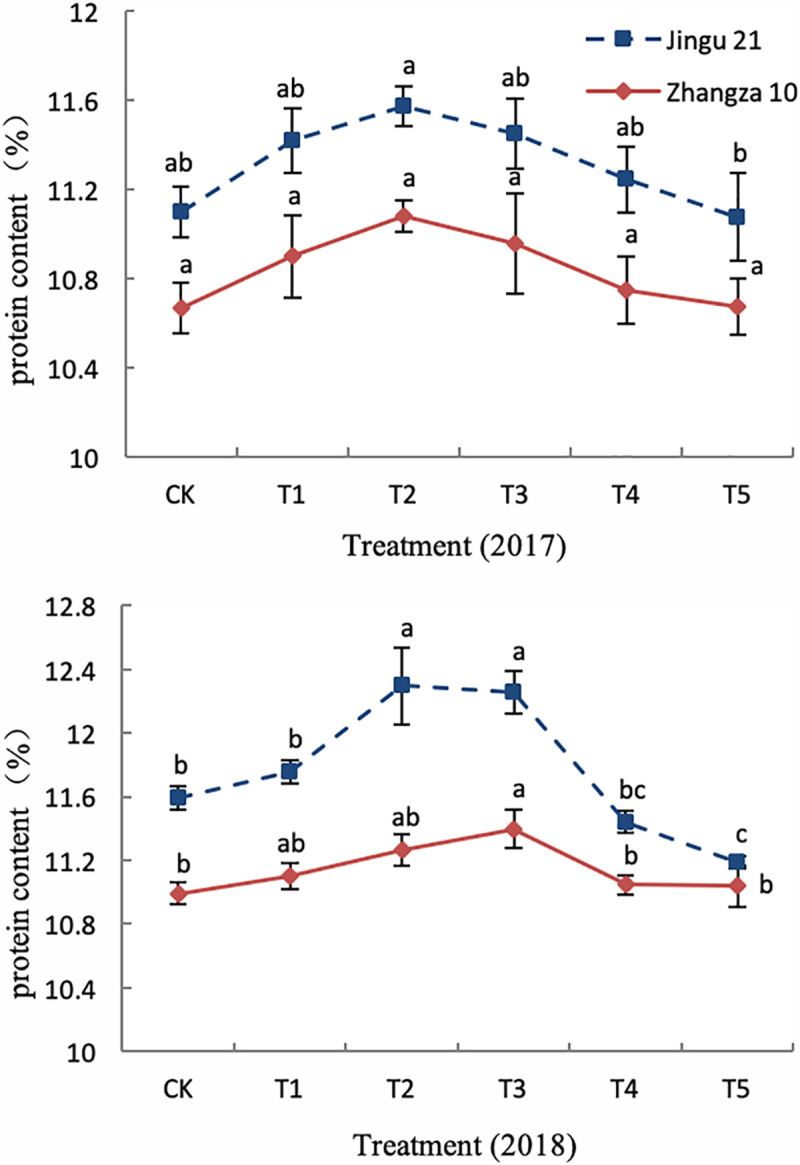
Effect of humic acid on protein content of foxtail millet in the years 2017 and 2018. CK, T1, T2, T3, T4, and T5 on the x-axis represent HA concentrations (0 mg L^-1^, 50 mg L^-1^, 100 mg L^-1^, 200 mg L^-1^, 300 mg L^-1^, and 400 mg L^-1^); y-axis represents protein content. Lowercase letters in the same column indicate significant differences at the 0.05 level.

As HA concentration increased, the protein content of Jingu21 and Zhangza10 grains initially increased and then decreased, in both 2017 and 2018 (Fig 2). Results of the membership function and comprehensive evaluation are shown in Table 3, and reveal an initial increase and subsequent decrease in nutrient uptake and yield in foxtail millet treated with HA. The order of action concentration for Jingu21 was T3 > T2 > T1 > T4 > T5, and for Zhangza10 was T2 > T3 > T1 > T4 > T5. The average membership function with T2 was 0.65 for Jingu21 and 0.80 for Zhangza10, and with T3 was 0.81 for Jingu21 and 0.69 for Zhangza10. The comprehensive analysis showed that the order of HA concentrations that improved foxtail millet yield and quality was T3 > T2 > T1 > T4 > T5.

**Table 3:** Membership function value of humic acid action on foxtail millet and comprehensive evaluation.

Cultivars	Treatment	Protein content (%)	P (g·kg^-1^)	K (g·kg^-1^)	Fe (mg·kg^-1^)	Mn (mg·kg^-1^)	Cu (mg·kg^-1^)	Zn (mg·kg^-1^)	Ca (mg·kg^-1^)	Mg (mg·kg^-1^)	Yield (kg·ha^-1^)	Mean value	Sort
Jingu21	CK	0.27	0.00	0.81	0.10	0.78	0.00	0.00	0.82	0.00	0.34	0.31	6
	T1	0.57	0.50	0.61	0.04	0.98	0.57	0.15	1.00	0.73	0.00	0.51	3
	T2	1.00	0.40	0.91	0.00	1.00	1.00	0.23	0.56	0.99	0.47	0.65	2
	T3	0.90	1.00	1.00	0.63	0.95	0.39	0.96	0.25	1.00	1.00	0.81	1
	T4	0.26	0.00	0.00	0.72	0.00	0.50	1.00	0.23	0.72	0.87	0.43	4
	T5	0.00	0.07	0.63	1.00	0.09	0.46	0.16	0.00	0.46	0.29	0.32	5
Zhangza10	CK	0.00	0.00	0.36	0.00	1.00	0.00	0.03	0.58	0.00	0.40	0.24	5
	T1	0.49	0.50	0.00	0.23	0.76	0.55	0.62	1.00	0.52	0.36	0.50	3
	T2	0.98	1.00	1.00	0.29	0.33	1.00	1.00	0.76	0.78	0.82	0.80	1
	T3	1.00	0.55	0.61	0.58	0.02	0.65	0.90	0.65	1.00	1.00	0.69	2
	T4	0.19	0.36	0.29	0.69	0.23	0.58	0.00	0.00	0.35	0.77	0.35	4
	T5	0.08	0.33	0.07	1.00	0.00	0.43	0.00	0.04	0.13	0.00	0.21	6

CK, T1, T2, T3, T4 and T5 represent HA concentrations (0 mg L^-1^, 50 mg L^-1^, 100 mg L^-1^, 200 mg L^-1^, 300 mg L^-1^ and 400 mg L^-1^).

### DEG analysis based on mRNA sequencing data

**Fig 3:**
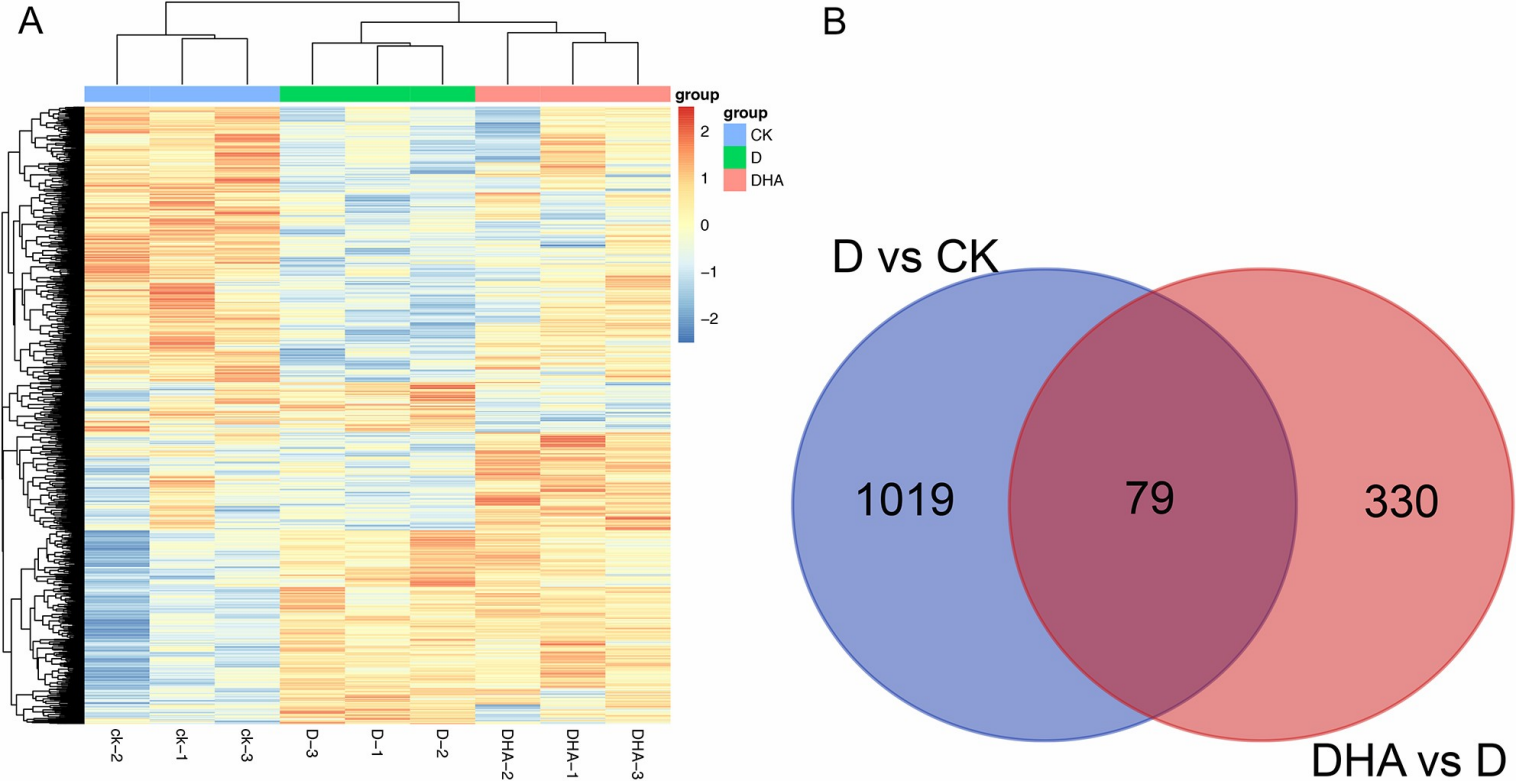
Differential gene expression in foxtail millet after HA treatment. A) heat map for DEGs among different groups; different colors represent different groups; values at *p* < 0.05 and |log2FC| > 0.585 were considered significantly different. B) Venn plot analysis for genes in all samples.

Sequencing data identified 453 genes that were up- regulated and 645 that were down-regulated between groups D and CK. Meanwhile, a total of 272 up-regulated and 137 down-regulated genes were identified between groups D and DHA. A heat map for the union of the two groups is shown in Fig 3A, and indicates the relative expression values of DEGs among samples in different groups. Furthermore, a Venn plot analysis was performed on all DEGs (Fig 3B), and revealed 79 common DEGs in both D vs. CK and D vs. DHA. Meanwhile, 1019 and 330 DEGs were identified for D vs. CK and D vs. DHA, respectively. Three hundred and thirty DEGs between groups D and DHA were selected for further investigation into the molecular mechanisms that underlie the effect of HA on dry matter and nutrient accumulation in foxtail millet.

### PPI network and module investigation

**Fig 4:**
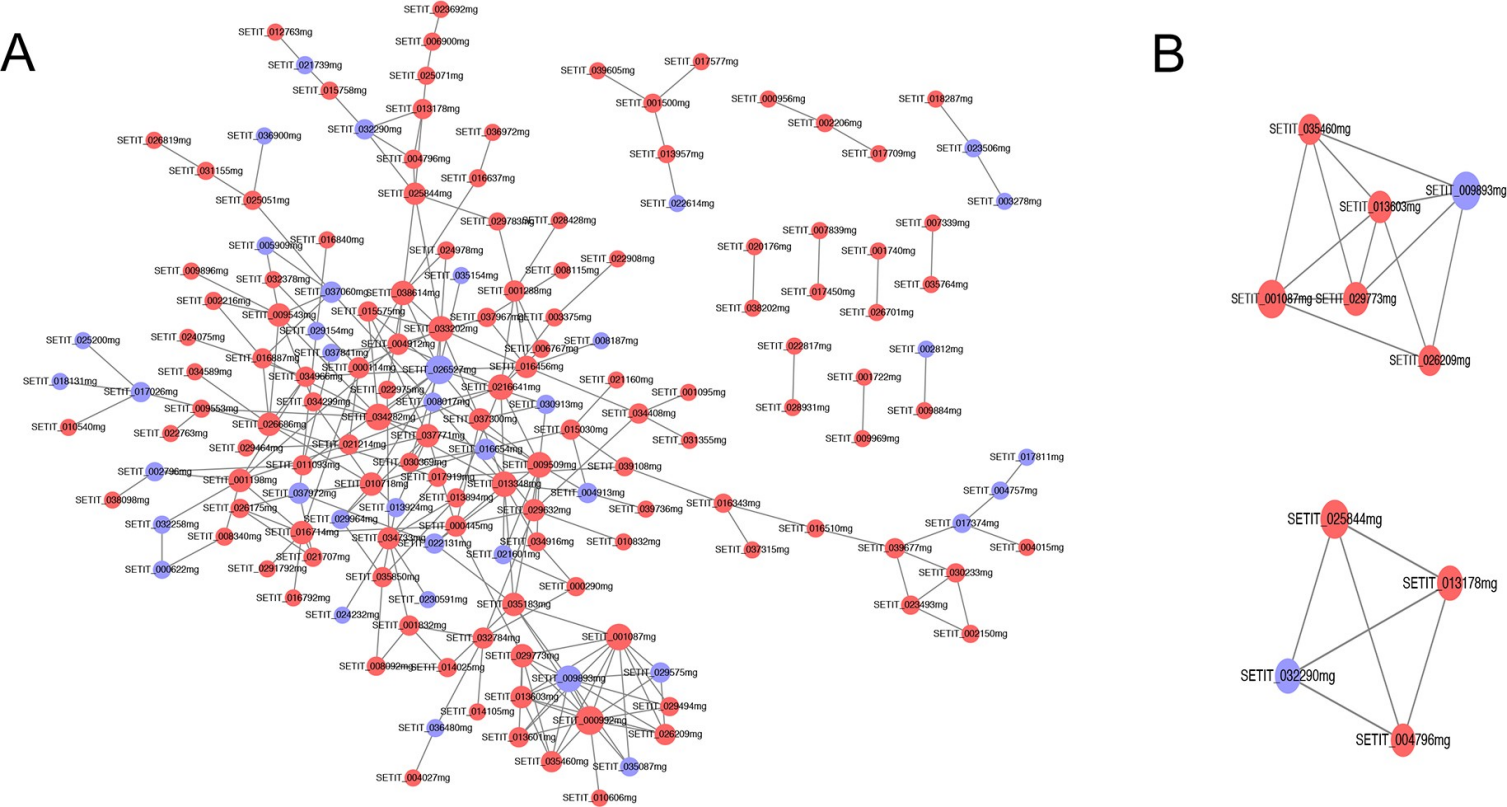
Protein-protein interaction network of differentially expressed genes. A, PPI network constructed from DEGs between groups D and DHA. B, two modules obtained from the PPI network. Red circles represent up-regulated DEGs; purple circles represent down-regulated DEGs. Gray lines represent protein interaction relationships. The larger the node, the higher the degree of interaction.

A PPI network was constructed based on DEGs between groups D and DHA (Fig 4A) with a combined score of 0.4. Moreover, with score ≥ 0.4, two modules, A (score = 4.8, six nodes, twelve interactions) and B (score = 4, four nodes, six interactions), were found to form the current PPI network (Fig 4B). Characteristic genes with top ten degrees were within the PPI network (such as SETIT_026527mg), module A (such as SETIT_001087MG), and module B (such as SETIT_025844mg).

### Enrichment analysis based on DEGs

**Fig 5:**
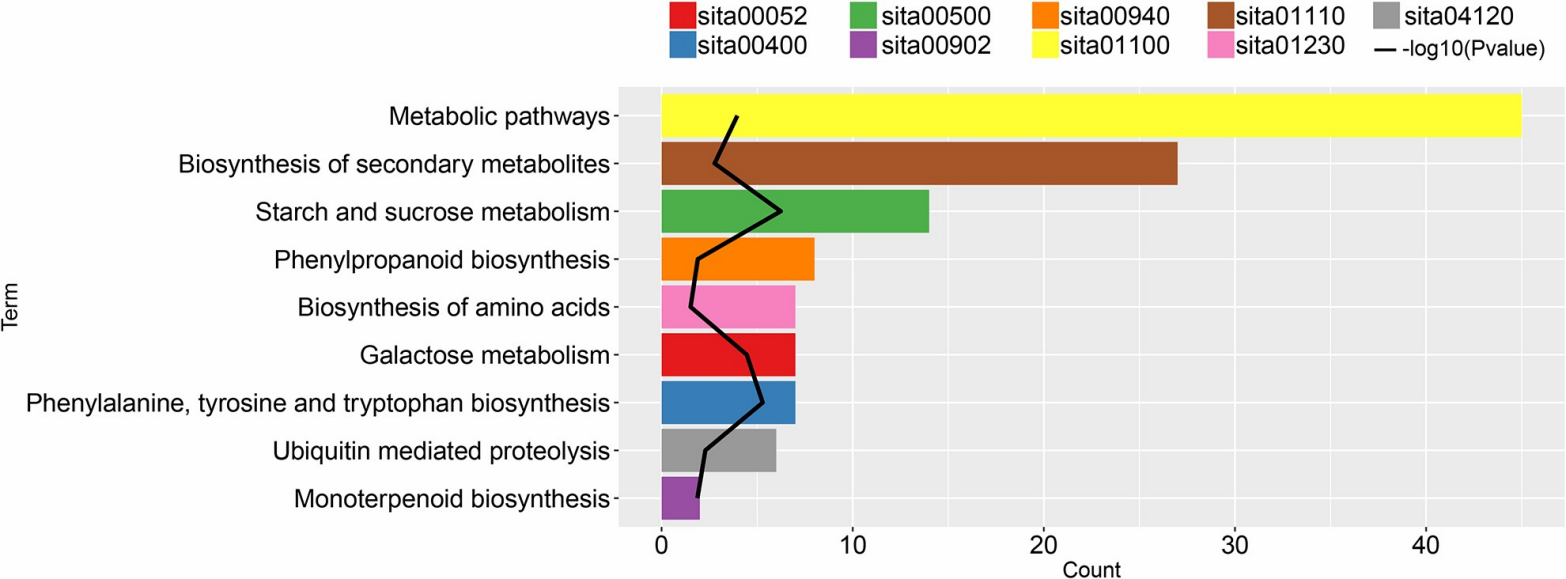
Pathway enrichment analysis of differentially expressed genes between groups D and DHA. Y-axis represents pathway annotation information; x-axis represents number of genes enriched in the pathway. The -log10 (*p*-value) is represented as an inflection point of the broken line.

A KEGG pathway enrichment analysis was performed on DEGs between groups D and DHA (Fig 5). Results show that the DEGs are mainly enriched in metabolic pathways (sita01100, genes: SETIT_009509mg, SETIT_017919mg, and SETIT_021617mg), secondary metabolite biosynthesis (sita01110, genes: SETIT_014105mg, SETIT_017811mg, and SETIT_022131mg), and starch and sucrose metabolism (sita00500, genes: SETIT_009543mg, SETIT_034299mg, and SETIT_009543mg).

### Gene expression analysis

**Fig 6:**
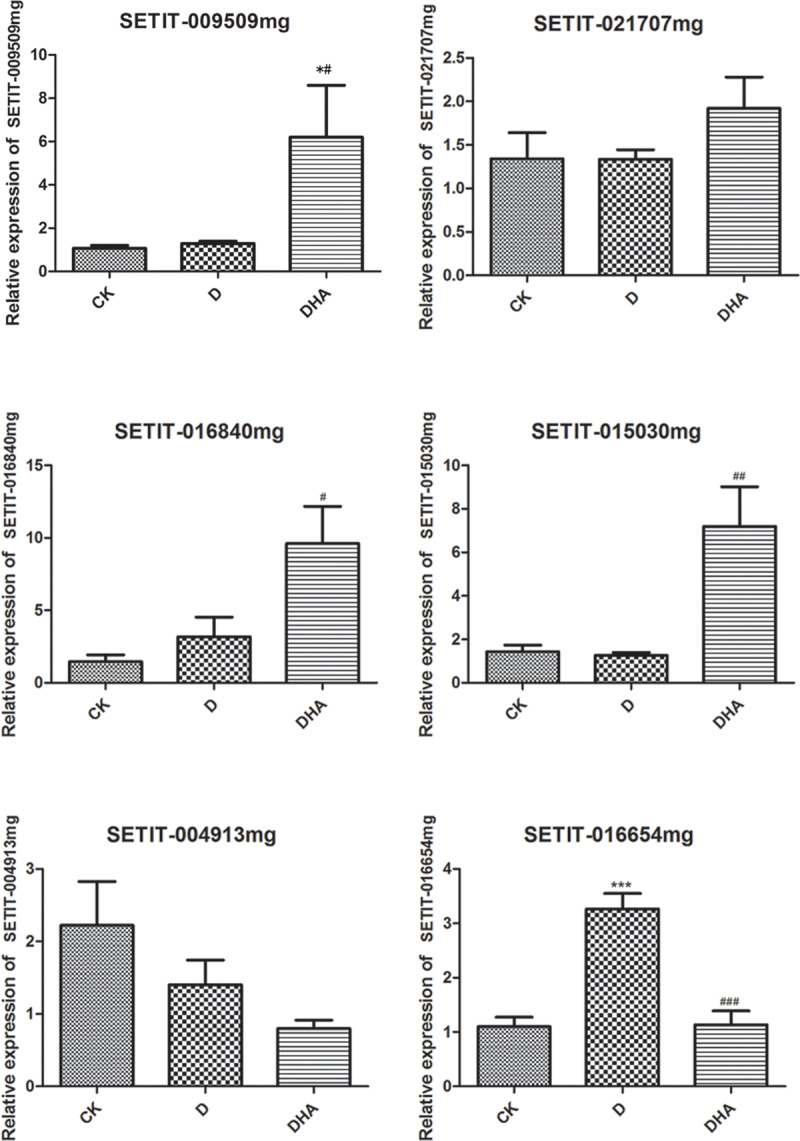
RT-qPCR analysis of gene expression in different HA treatment groups. X-axis represents different groups; y-axis represents relative gene expression. *, *p* < 0.05 when compared with the CK group; #, *p* < 0.05 when compared with the D group.

Expression of human SETIT_009509mg, SETIT_021707mg, SETIT_016840mg, SETIT_015030mg, SETIT_004913mg, and SETIT_016654mg genes was analyzed by RT-qPCR. Results show that SETIT_016654mg was significantly up-regulated in group D compared with the CK group (*p* < 0.05). Moreover, SETIT_021707mg, SETIT_016840mg, and SETIT_015030mg were significantly up-regulated, while SETIT_004913mg and SETIT_016654mg were significantly down-regulated in the DHA group compared with group D (all *p* < 0.05) (Fig 6). Finally, SETIT-009509mg was significantly up-regulated in the DHA group compared to groups CK and D (all *p* < 0.05).

## Discussion

Organic HA contains macronutrients and micronutrients, growth-promoting substances, vitamins, and beneficial microorganisms, all of which are important for maximizing plant yield. Previous research showed that HA treatment resulted in an increase in grain yield in wheat [27]. For the two varieties of millet in the present study, there was no difference in P content between CK and the T4 treatment, whereas other concentrations of HA resulted in an increase in P content. This increase in phosphorus after HA treatment could be due its forming a complex with iron (Fe) [28]. Some trace elements, including Fe, have low solubility in soil at higher pH values, which can result in Fe deficiency in plants. The addition of HA can reduce the soil pH [29], which in turn would not only promote the growth of crops, but could also increase nutrient absorption from the soil [27]. Humic acid can either activate or inhibit enzyme activity by promoting the absorption of underground soil nutrients by the plant, thus altering cell membrane permeability, which in turn leads to protein synthesis and stimulates growth, ultimately increasing the yield.

In the current study, HA was found to alleviate drought stress in foxtail millet. More specifically, treatment with 100–200 mg L^-1^ HA had a positive effect on underground dry weight and root-shoot ratio., and also increased the yield. There was a strong positive correlation between root-shoot ratio and yield (*p* < 0.01). An increase in underground dry weight is an indication of enhanced root growth, which could be a good way to alleviate moisture loss in the soil [30]. It has been shown that addition of HA to soil increases proliferation and branching of root hairs [31], which could in part explain the enhanced nutrient absorption and increased yield observed in the present study. HA treatment had little influence on ear length, ear diameter or ear yardage of millet, which could be because the HA was applied during the jointing and filling stages, when these ear characteristics are already defined [32].

Drought stress is one of the major abiotic stresses known to affect crop production worldwide. In order to understand the mechanism by which plants cope with water deficiency, it is necessary to study natural drought-tolerant plants and identify the molecular mechanisms that underlie their drought stress tolerance. The biological function of HA is commonly studied in terms of regulation at either the gene or molecular level [33]. In the present study, 330 DEGs were identified between groups D and DHA, and were found to be enriched mainly in metabolic pathways (SETIT_009509mg, SETIT_017919mg, and SETIT_021617mg), secondary metabolite biosynthesis (SETIT_014105mg, SETIT_017811mg, and SETIT_022131mg), and starch and sucrose metabolism (SETIT_009543mg, SETIT_034299mg, and SETIT_009543mg) [34, 35], indicating that these pathways are dominant when the plant is under drought stress. The expression of six genes, which are involved in important metabolic pathways and with high degrees of interaction in a PPI network, were verified by RTqPCR. Of these, SETIT_016654mg was significantly up-regulated in group D compared to groups CK and DHA (*p* < 0.0001). SETIT_016654mg encodes arginine decarboxylase 2 (ADC2), which has been shown to be induced by drought stress in *Arabidopsis thaliana* [36]. Under high permeability conditions, such as after treatment with mannitol, ADC activity was found to be increased in the leaves and roots of wheat. Furthermore, putrescine is known to play an important role in salt tolerance in plants [37], and levels of putrescine and spermine were increased in the leaves and roots of plants treated with mannitol,.

## Conclusions

In conclusion, HA at a concentration of 100 mg L^-1^ can increase the yield and protein and mineral content of foxtail millet grain under drought conditions. Moreover, SETIT_016654mg may play an important role in the effect of HA on foxtail millet by regulating metabolic pathways. This study identified the treatment dose of HA that should be used in millet under drought conditions, and lays the foundation for research into the molecular mechanisms that underlie the alleviating effects of HA on foxtail millet under drought conditions.

## Supporting information

S1 Table Primers used for RT-qPCR. (DOCX)

S2 Table Effect of humic acid on growth indicators of foxtail millet in the years 2017 and 2018. (DOCX)

## References

[1] Tsai KJ, Lu MJ, Yang K, et al Assembling the Setaria italica L. Beauv. genome into nine chromosomes and insights into regions affecting growth and drought tolerance. *Scientific Reports* 2016; 6: 35076 10.1038/srep35076 27734962 PMC5062080

[2] Li H and Perret S. Irrigation Management Reform in Northern China: Case Studies in Shanxi Province. *Irrigation and Drainage* 2015; 64: 193–204.

[3] Kalaichelvi K, Chinnusamy C and Swaminathan AA. EXPLOITING THE NATURAL RESOURCE—LIGNITE HUMIC ACID IN AGRICULTURE—A REVIEW. *Agricultural Reviews* 2006; 27: 276–283.

[4] Fallahi H, Ghorbany M, Aghhavanishajari M, et al Qualitative response of roselle to planting methods, humic acid application, mycorrhizal inoculation and irrigation management. *Journal of Crop Improvement* 2017; 31: 192–208.

[5] Hseu Y-C, Kumar KS, Chen C-S, et al Humic acid in drinking well water induces inflammation through reactive oxygen species generation and activation of nuclear factor-κB/activator protein-1 signaling pathways: a possible role in atherosclerosis. *Toxicology and applied pharmacology* 2014; 274: 249–262. 10.1016/j.taap.2013.11.002 24239652

[6] Vaccaro S, Ertani A, Nebbioso A, et al Humic substances stimulate maize nitrogen assimilation and amino acid metabolism at physiological and molecular level. *Chemical and Biological Technologies in Agriculture* 2015; 2: 5.

[7] Parađiković N, Vinković T, Vinković Vrček I, et al Effect of natural biostimulants on yield and nutritional quality: an example of sweet yellow pepper (Capsicum annuum L.) plants. *Journal of the Science of Food and Agriculture* 2011; 91: 2146–2152. 10.1002/jsfa.4431 21538369

[8] El-Nemr M, El-Desuki M, El-Bassiony A, et al Response of growth and yield of cucumber plants (Cucumis sativus L.) to different foliar applications of humic acid and bio-stimulators. *Australian Journal of Basic and Applied Sciences* 2012; 6: 630–637.

[9] Maji D, Misra P, Singh S, et al Humic acid rich vermicompost promotes plant growth by improving microbial community structure of soil as well as root nodulation and mycorrhizal colonization in the roots of Pisum sativum. *Applied soil ecology* 2017; 110: 97–108.

[10] Celik H, Katkat AV, Aşık BB, et al Effect of foliar-applied humic acid to dry weight and mineral nutrient uptake of maize under calcareous soil conditions. *Communications in soil science and plant analysis* 2010; 42: 29–38.

[11] Olaetxea M, Mora V, Bacaicoa E, et al ABA-regulation of root hydraulic conductivity and aquaporin gene-expression is crucial to the plant shoot rise caused by rhizosphere humic acids. *Plant Physiology* 2015: pp. 00596.02015. 10.1104/pp.15.00596 PMC4677878 26450705

[12] Kuşvuran VSA and Babat S. The effect of different humic acid fertilization on yield and yield components performances of common millet (Panicum miliaceum L.). *Scientific Research and Essays* 2011; 6: 663–669.

[13] Peng Y, Shen C, Wang L, et al A novel classification method based on membership function In: *Medical Imaging 2011*: *Image Processing* 2011, p.79623L. International Society for Optics and Photonics.

[14] Andrews S. Babraham bioinformatics-FastQC a quality control tool for high throughput sequence data. *URL: https://www.bioinformatics.babraham.ac.uk/projects/fastqc/ (accessed 0612 2018)* 2015.

[15] Trapnell C, Pachter L and Salzberg S. TopHat: discovering splice junctions with RNA-Seq. Bioinformatics 25, 1105e1111 2009. 19289445 10.1093/bioinformatics/btp120 PMC2672628

[16] Kersey PJ, Allen JE, Allot A, et al Ensembl Genomes 2018: an integrated omics infrastructure for non-vertebrate species. *Nucleic acids research* 2017; 46: D802–D808. 10.1093/nar/gkx1011 PMC5753204 29092050

[17] Liao Y, Smyth GK and Shi W. featureCounts: an efficient general purpose program for assigning sequence reads to genomic features. *Bioinformatics* 2013; 30: 923–930. 10.1093/bioinformatics/btt656 24227677

[18] Nikolayeva O and Robinson MD. edgeR for differential RNA-seq and ChIP-seq analysis: an application to stem cell biology *Stem Cell Transcriptional Networks*. Springer, 2014, pp.45–79. 10.1007/978-1-4939-0512-6_3 24743990

[19] Robinson MD, McCarthy DJ and Smyth GK. edgeR: a Bioconductor package for differential expression analysis of digital gene expression data. *Bioinformatics* 2010; 26: 139–140. 10.1093/bioinformatics/btp616 19910308 PMC2796818

[20] Oliveros JC. VENNY. An interactive tool for comparing lists with Venn Diagrams. 2007.

[21] Shannon P, Markiel A, Ozier O, et al Cytoscape: a software environment for integrated models of biomolecular interaction networks. *Genome research* 2003; 13: 2498–2504. 10.1101/gr.1239303 14597658 PMC403769

[22] Bandettini WP, Kellman P, Mancini C, et al MultiContrast Delayed Enhancement (MCODE) improves detection of subendocardial myocardial infarction by late gadolinium enhancement cardiovascular magnetic resonance: a clinical validation study. *Journal of Cardiovascular Magnetic Resonance* 2012; 14: 83 10.1186/1532-429X-14-83 23199362 PMC3552709

[23] Bader GD HC. An automated method for finding molecular complexes in large protein interaction networks. *BMC Bioinformatics* 2003; 13. 10.1186/1471-2105-4-2 PMC149346 12525261

[24] Kanehisa M and Goto S. KEGG: Kyoto Encyclopedia of Genes and Genomes. *Nucleic Acids Research* 2000; 28: 27–30. 10.1093/nar/28.1.27 10592173 PMC102409

[25] Wu J, Mao X, Cai T, et al KOBAS server: a web-based platform for automated annotation and pathway identification. *Nucleic acids research* 2006; 34: W720–W724. 10.1093/nar/gkl167 16845106 PMC1538915

[26] Livak KJ ST. Analysis of Relative Gene Expression Data Using Real-Time Quantitative PCR and the 2(-Delta Delta C(T))Method. *METHODS* 2001; Dec;25(4): 402–408. 10.1006/meth.2001.1262 11846609

[27] Khan RU, Khan MZ, Khan A, et al Effect of humic acid on growth and crop nutrient status of wheat on two different soils. *Journal of Plant Nutrition* 2018; 41: 453–460. 10.1080/01904167.2017.1385807

[28] David PP, Nelson PV and Sanders DC. A humic acid improves growth of tomato seedling in solution culture. *Journal of Plant Nutrition* 1994; 17: 173–184.

[29] Nandakumar R, Science ASoS, Agricultural Chemistry TNAUC, India, et al Effect of lignite humic acid on soil nutrient availability at different growth stages of rice grown on Vertisols and Alfisols. *Acta Agronomica Hungarica* 2015; 52: 227–235.

[30] Stevenson FJ. Humus Chemistry: Genesis, Composition, Reactions, 2nd Edition. *Soil Science* 1982; 135: 129–130.

[31] Atiyeh RM, Lee S, Edwards CA, et al The influence of humic acids derived from earthworm-processed organic wastes on plant growth. *Bioresource Technology*; 84: 7–14. 10.1016/s0960-8524(02)00017-2 12137272

[32] Fan GY, Zhao ZH, Yuan JC, et al Study the Relationship between Anatomical Structure and Biology Characters of Hybrid Millet Zhangzagu 3 hao. *Acta Agriculturae Boreali-Sinica* 2014; 01: 114–119.

[33] Tebbe CC and Vahjen W. Interference of humic acids and DNA extracted directly from soil in detection and transformation of recombinant DNA from bacteria and a yeast. *Appl Environ Microbiol* 1993; 59: 2657–2665. 7690221 10.1128/aem.59.8.2657-2665.1993 PMC182335

[34] Ma YH, Li SY, Lin H, et al Bioinformatics Analysis of Microarray Data to Reveal Novel Genes Related to Cold-Resistance of Maize. *Russian Journal of Plant Physiology*; 65: 278–285.

[35] Cheng YQ, Liu JF, Yang X, et al RNA-seq Analysis Reveals Ethylene-Mediated Reproductive Organ Development and Abscission in Soybean (Glycine maxL. Merr.). *Plant Molecular Biology Reporter* 2013; 31: 607–619.

[36] Alcázar R, Cuevas JC, Patron M, et al Abscisic acid modulates polyamine metabolism under water stress in Arabidopsis thaliana. *Physiologia Plantarum* 2006; 128: 448–455.

[37] Foster SA and Walters DR. Polyamine concentrations and arginine decarboxylase activity in wheat exposed to osmotic stress. *Physiologia Plantarum* 1991; 82: 185–190.

