# Supplementary material for: An investigation into the beneficial effects and molecular mechanisms of humic acid on foxtail millet under drought conditions
Source: PLoS One. 2020 Jun 2;15(6):e0234029. doi: 10.1371/journal.pone.0234029 (PMC7266348; doi:10.1371/journal.pone.0234029)
Supplement: S1 Table — (DOCX) [file pone.0234029.s001.docx]

**Supplementary Table S1. Primers used for RT-qPCR**

| **Primer** | **Sequence** |
| --- | --- |
| SETIT_009509mg | Forward: 5′- GCCTGGACTATGGGTTCAAGC -3′  Reverse: 5′- CCTGGGTCACGGTGTTCTTC ′ |
| SETIT_021707mg | Forward: 5′- CTTCCTTGCCGTCTTTCGC -3′  Reverse: 5′- CTCCTGCCTGGTGCTTCTC -3′ |
| SETIT_016840mg | Forward: 5′- CCGATCAGGTTCCTACCGC -3′  Reverse: 5′- GCCCAAGTCATCCCAAAGC -3′ |
| SETIT_015030mg | Forward: 5′- CCGTCATCACCGACCTCAA -3′  Reverse: 5′- CCGCCTCCCATCAGTAGAA -3′ |
| SETIT_004913mg | Forward:5’- GTTTTGTTCGCCAATCCTTT -3’  Reverse: 5’- TGCGGTTTACACCAGCATCA -3’ |
| SETIT_016654mg | Forward:5’- TCCCGATCAAGGTGAACCA -3’  Reverse: 5’- AGCCGTTGCAGACCAAGTAAG -3’ |
| Actin | Forward: 5′- GGCAAACAGGGAGAAGATGA -3′  Reverse: 5′- GAGGTTGTCGGTAAGGTCACG -3′ |
