# Supplementary material for: An investigation into the beneficial effects and molecular mechanisms of humic acid on foxtail millet under drought conditions
Source: PLoS One. 2020 Jun 2;15(6):e0234029. doi: 10.1371/journal.pone.0234029 (PMC7266348; doi:10.1371/journal.pone.0234029)
Supplement: S2 Table — (DOCX) [file pone.0234029.s002.docx]

**Supplementary Table 2 Effects of humic acid on growth indicators of foxtail millet in the year 2017 and 2018**

| Cultivars | Treatment | 2017 | | | 2018 | | |
| --- | --- | --- | --- | --- | --- | --- | --- |
|  |  | Aboveground dry weight (g) | Underground dry weight (g) | Root-shoot ratio (%) | Aboveground dry weight (g) | Underground dry weight (g) | Root-shoot ratio (%) |
| Jingu 21 | CK | 30.00±0.51d | 4.94±0.09b | 16.50±0.56b | 24.57±0.29c | 3.20±0.02bc | 13.02±0.13bc |
|  | T1 | 38.78±0.64a | 5.44±0.16ab | 14.06±0.64cd | 30.18±0.48a | 3.06±0.10c | 10.14±0.46d |
|  | T2 | 36.44±0.53b | 5.67±0.19a | 15.57±0.72bcd | 29.04±0.62a | 3.43±0.04bc | 11.81±0.17cd |
|  | T3 | 29.78±0.80d | 5.89±0.11a | 19.82±0.88a | 26.80±0.38b | 4.50±0.30a | 17.01±0.90a |
|  | T4 | 33.78±0.68c | 5.39±0.20ab | 15.94±0.32bc | 26.45±0.40b | 4.26±0.21a | 15.87±0.62a |
|  | T5 | 35.67±0.51bc | 4.96±0.12b | 13.90±0.32d | 26.49±0.37b | 3.69±0.20b | 13.92±0.72b |
| Zhangza 10 | CK | 15.83±0.29c | 3.59±0.05c | 22.64±0.11b | 18.50±0.35a | 3.32±0.07b | 18.00±0.64c |
|  | T1 | 16.33±0.51bc | 3.61±0.20c | 22.21±1.83b | 18.45±0.71a | 3.31±0.14b | 17.93±0.45c |
|  | T2 | 12.56±0.31d | 4.67±0.19b | 37.28±2.38a | 16.62±0.43b | 3.62±0.10ab | 21.81±0.27a |
|  | T3 | 18.89±0.80a | 5.17±0.15a | 27.51±1.86b | 19.09±0.47a | 4.04±0.11a | 21.23±1.08ab |
|  | T4 | 17.56±0.48ab | 4.56±0.13b | 26.02±1.34b | 19.13±0.32a | 3.62±0.16ab | 18.92±0.70bc |
|  | T5 | 18.89±0.48a | 4.33±0.19b | 22.99±1.33b | 18.04±0.52ab | 3.21±0.21b | 17.78±1.15c |

**Note**: CK, T1, T2, T3, T4 and T5 represented different concentration of HA (0 mg L^-1^, 50 mg L^-1^, 100 mg L^-1^, 200 mg L^-1^, 300 mg L^-1^ and 400 mg L^-1^); different lowercases in the same column indicated significant differences at 0.05 level.
